# Development and Characterization of Curcumin-Silver Nanoparticles as a Promising Formulation to Test on Human Pterygium-Derived Keratinocytes

**DOI:** 10.3390/molecules27010282

**Published:** 2022-01-03

**Authors:** Gianmarco Stati, Francesco Rossi, Thithawat Trakoolwilaiwan, Le Duc Tung, Stefanos Mourdikoudis, Nguyễn Thi Kim Thanh, Roberta Di Pietro

**Affiliations:** 1 Department of Medicine and Ageing Sciences, G. d’Annunzio University of Chieti-Pescara, Via dei Vestini, 31, 66100 Chieti, Italy; 2 UCL Healthcare Biomagnetic & Nanomaterials Laboratories, The Royal Institution of Great Britain, 21 Albemarle Street, London W1S 4BS, UK; francesco.rossi@unive.it (F.R.); t.trakoolwilaiwan@ucl.ac.uk (T.T.); t.le@ucl.ac.uk (L.D.T.); mourdikt@vscht.cz (S.M.); 3 Biophysics Group, Department of Physics & Astronomy, University College London, Gower Street, London WC1E 6BT, UK; 4 Department of Molecular Sciences and Nanosystems, Ca’ Foscari University, Via Torino, 155/b, 30170 Venice, Italy; 5 Healthy Infrastructure Research Group, University College London, Gower Street, London WC1E 6BT, UK; 6 Department of Inorganic Chemistry, University of Chemistry and Technology Prague, Technicka 5, 166 28 Prague, Czech Republic

**Keywords:** curcumin, silver, pterygium, keratinocytes, nanoparticles, green synthesis

## Abstract

Pterygium is a progressive disease of the human eye arising from sub-conjunctival tissue and extending onto the cornea. Due to its invasive growth, pterygium can reach the pupil compromising visual function. Currently available medical treatments have limited success in suppressing efficiently the disease. Previous studies have demonstrated that curcumin, polyphenol isolated from the rhizome of *Curcuma longa*, induces apoptosis of human pterygium fibroblasts in a dose- and time-dependent manner showing promising activity in the treatment of this ophthalmic disease. However, this molecule is not very soluble in water in either neutral or acidic pH and is only slightly more soluble in alkaline conditions, while its dissolving in organic solvents drastically reduces its potential use for biomedical applications. A nanoformulation of curcumin stabilized silver nanoparticles (Cur-AgNPs) seems an effective strategy to increase the bioavailability of curcumin without inducing toxic effects. In fact, silver nitrates have been used safely for the treatment of many ophthalmic conditions and diseases for a long time and the concentration of AgNPs in this formulation is quite low. The synthesis of this new compound was achieved through a modified Bettini’s method adapted to improve the quality of the product intended for human use. Indeed, the pH of the reaction was changed to 9, the temperature of the reaction was increased from 90 °C to 100 °C and after the synthesis the Cur-AgNPs were dispersed in Borax buffer using a dialysis step to improve the biocompatibility of the formulation. This new compound will be able to deliver both components (curcumin and silver) at the same time to the affected tissue, representing an alternative and a more sophisticated strategy for the treatment of human pterygium. Further in vitro and in vivo assays will be required to validate this formulation.

## 1. Introduction

Pterygium, a wing-shaped fibro-vascular overgrowth arising from the white and clear part of the eye (sclera), is able to spread through the corneoscleral junction and to invade the cornea. Due to its progressive growth, pterygium can impair the visual function, in rare cases leading to blindness [1]. The current treatment of this ophthalmic disease is a surgical removal of the lesion. In the years, different surgical procedures have been developed to treat this condition, but all available methods can lead to a relapse of the disease which always consists in a faster growing and more aggressive form of pterygium [1]. Several medical therapies, such as the administration of thiotepa, 5-fluoruracil, mitomycin C, VEGF inhibitors and beta irradiation, have been used. These treatments have not led to a consistent reduction in the relapse cases. Several research groups reported a different percentage of relapses, corneal scarring and side effects respectively, due to surgical procedure and drugs utilized [2,3,4].

Recent in vitro studies carried out in our research lab have demonstrated that an alcoholic extract of 1.3% *Curcuma longa* induces apoptosis of keratinocytes derived from explants of human pterygium [5]. Other authors have demonstrated that curcumin, the main active polyphenol isolated from the rhizome of *Curcuma longa*, induces apoptosis of human pterygium fibroblasts in a dose- and time-dependent manner [6]. Curcumin has been for centuries used in traditional medicine by virtue of its beneficial properties due to anti-inflammatory, anti-angiogenic, anti-carcinogenic, and antioxidant effects [7]. Still, it has not yet reached the clinical stage due to its low solubility in water and low bioavailability [8]. Various studies have reported that the bioavailability and stability of curcumin could be increased by conjugating it with metal nanoparticles (NPs) [9].

Metal NPs can be a versatile carrier for the delivery of biomolecules [10], which have numerous applications either as medical devices or anticancer treatments [11,12]. The use of silver salts for the treatment of ophthalmic infections or angiogenic conditions dates back to long time ago [13]. In recent years, silver NPs (AgNPs) have seen numerous applications in conjunction with biomolecules [14,15,16]. However, there are still concerns regarding the in vivo toxicity of AgNPs. In fact, though silver nitrate has been widely used all over the world for the treatment of perinatal eye infection with no evidence of any toxicity [17], it has been reported that AgNPs are able not only to induce inflammation and oxidative stress at the site of exposure, but also to cross various biological barriers and enter the systemic circulation [18]. On the other hand, other studies have demonstrated that AgNPs could be relatively safe when administered to oral mucosa, eye and skin of the animal models for short periods of time and at doses up to 1 mg/kg [19,20].

Although various active molecules have been designed to associate with nanocarriers to overcome ocular barriers and intimately interact with specific ocular tissues [21], to our knowledge there are no reports regarding the use of curcumin-based nanocarriers for the treatment and prevention of human pterygium.

Thus, based on current literature and our previous observations, the goal of this study was to achieve an innovative formulation to test in vitro on human keratinocytes derived from pterygium explants. To this aim, we have synthesized curcumin stabilized silver NPs (Cur-AgNPs) with the purpose of exploiting the features provided by both curcumin and silver synergistically reinforcing their effectiveness on our biological target. Since this conjugate is destined for human medical use, we explored a number of modifications in the synthesis method described by Bettini et al. [22] in order to achieve a formulation compatible with intended use in humans.

## 2. Results

We first carefully analyzed the Bettini’s protocol to obtain silver NPs capped by the natural compound (1E, 6E)-1,7-bis(4-hydroxy-3-methoxyphenyl)-1,6-heptadiene-3,5-diene), also known as curcumin [22]. We then applied some changes to the green synthesis method to improve the quality of our nanocomposite intended for clinical use.

To overcome the disadvantage of lengthy reaction times, we carried out the synthesis of Cur-AgNPs using a reaction flask instead of a ventilated oven. The temperature of the reaction was increased from 90 °C (Bettini’s method) to 100 °C and the pH was carefully controlled to values slightly below 9 in the course of the reaction to achieve the highest possible reactivity of curcumin and silver, together with the lowest degree of alkaline decomposition [23]. Furthermore, after synthesis, a dialysis was applied to remove the residues of reaction, then we lowered the pH, and transferred the produced NPs from ultrapure water to a borax buffer solution. This procedure was necessary to move the NPs from the bi-distilled water used in the Bettini’s synthesis mixture to a more biocompatible buffer (borax), without compromising the stability of the particles. Indeed, the formulation in buffer was sufficiently concentrated to eliminate the risk of human cells to suffer from osmotic stress when exposed to it. The Cur-AgNPs obtained were characterized with a range of techniques to demonstrate the efficiency of the synthesis and the successful attachment of curcumin on their surface.

### 2.1. Study of the Size Distribution of Cur-AgNPs

**Figure 1:**
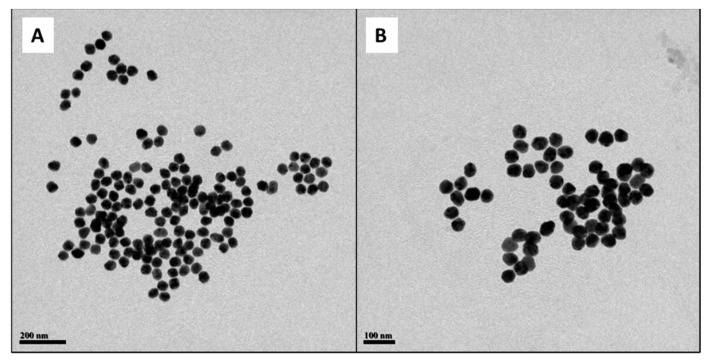
TEM images of Cur-AgNPs. (**A**) 7500× magnification and (**B**) 10,000× magnification. The images are the most representative among the five different batches produced with this synthesis.

To determine the mean diameter of the silver core of the Cur-AgNPs, several transmission electron microscopy (TEM) images were acquired (Figure 1). The images were then processed using an image analysis software (ImageJ) to measure the diameter of each one of the particles in different images.

**Figure 2:**
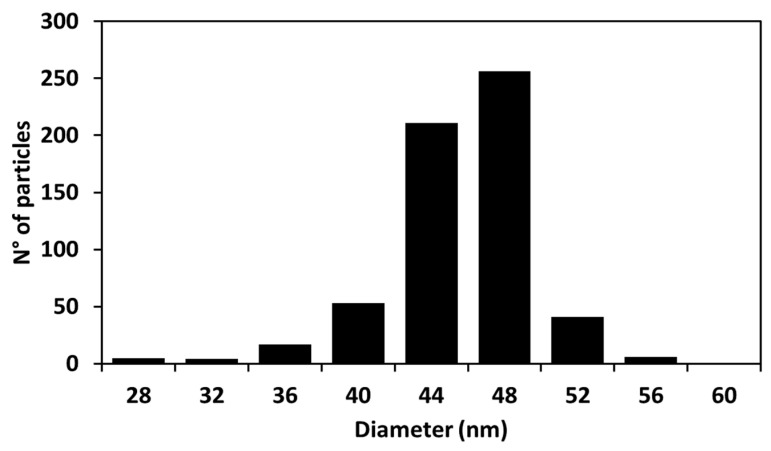
Histogram of the Cur-AgNPs diameters obtained from analyzing the TEM images with ImageJ software (the size of 605 particles was measured).

From the analysis of the distribution of diameters (Figure 2), it was found that our synthetized particles are monodispersed with an average diameter of 44.9 ± 2.2 nm.

**Figure 3:**
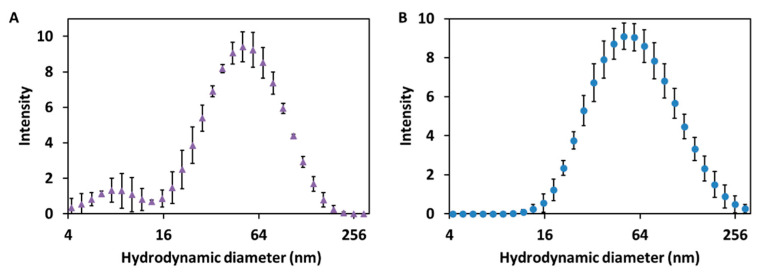
DLS analysis of the hydrodynamic diameters of Cur-AgNPs. (**A**) before dialysis and (**B**) after dialysis. The x axis representing the particle size is presented in logarithmic scale.

To complete the characterization of the size of the synthesized Cur-AgNPs, dynamic light scattering (DLS) analysis of their dispersion was performed before and after the dialysis step (Figure 3A,B).

The DLS analysis of the dispersion of Cur-AgNPs (Figure 3A,B) shows that the hydrodynamic diameter of the particles was consistent before and after dialysis. A mean size of 53.0 ± 4.0 nm was determined for the sample tested before dialysis, whereas after the dialysis process the corresponding average diameter was 55.3 ± 4.7 nm. From the comparison of the size distribution information obtained from the DLS studies before and after dialysis (Figure 3A,B) and the study of the TEM images (Figure 1 and Figure 2), a certain degree of discrepancy is noticed between the results derived from both techniques: DLS yielded a size of approximately 55 nm, while the mean size observed with TEM was 44.9 ± 2.2 nm. This discrepancy is generated by three main factors: (1) DLS technique measures the hydrodynamic diameter of the particles, thus the shell of curcumin and the volume of associated water directly influences their electrostatic charge [24]; (2) larger particles scatter more light compared to smaller particles so they tend to be overestimated by DLS [25]; and, finally, (3) the contrast of TEM images depends on the electron density of a material compared to the carbon mesh of the support, making the curcumin layer of the particles, that is thin and with a low contrast, almost impossible to capture with TEM imaging [26].

### 2.2. Study of the ζ-Potential and Optical Properties of Cur-AgNPs

**Figure 4:**
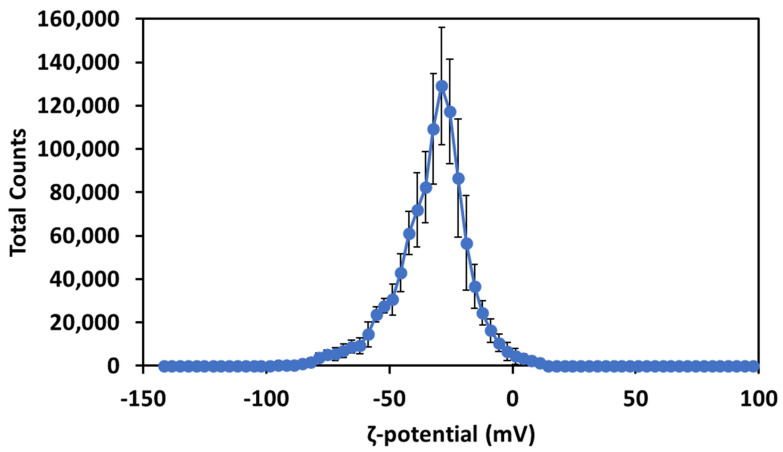
ζ-potential of Cur-AgNPs after dialysis.

The ζ-potential of the Cur-AgNPs (Figure 4) was investigated after the dialysis step.

The measurement of the ζ-potential showed that the presence of curcumin on the surface of the particles infers a negative electrostatic potential of −32.5 ± 0.5 mV and stabilizes electrostatically the particles. This surface charge is consistent with the potential of the carboxylic moieties of curcumin at pH 7 and is strong enough to grant an electrostatic stabilization to the Cur-AgNPs [27].

**Figure 5:**
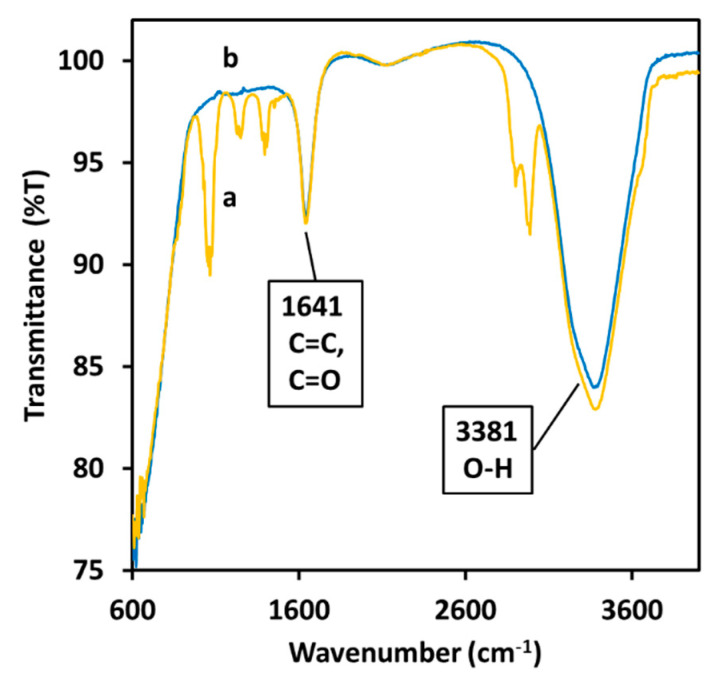
FTIR (Fourier-transform infrared spectroscopy) analysis of 10.6 μM curcumin in water (**a**) and of a dispersion of Cur-AgNPs in water (**b**).

The IR (infrared) absorption of the NPs dispersion was used to detect the presence of curcumin on the surface of the NPs after the dialysis. The spectrum of curcumin after the formation of the particles (Figure 5b) is less defined than its spectrum in water (Figure 5a).

The spectrum shows a large peak around 3381 cm^−1^ attributed to water molecules and alcoholic moieties being present in the sample. The sharp peak at 1641 cm^−1^ indicates the existence of carbon double bonds and carbon oxide double bond. The sharp double peak at 2750 cm^−1^ spotted in the spectrum of curcumin disappears after the synthesis of the NPs while the three peaks around 1250 cm^−1^ generated by the aromatic rings are almost completely quenched by the presence of the silver NPs. The weak peaks in the section between 1105–1281 cm^−1^ indicate the presence of carbon-oxygen single bonds.

Spectroscopic techniques were used to characterize the UV-visible absorption of the Cur-AgNPs and to further confirm the presence of curcumin on the surface of the NPs.

**Figure 6:**
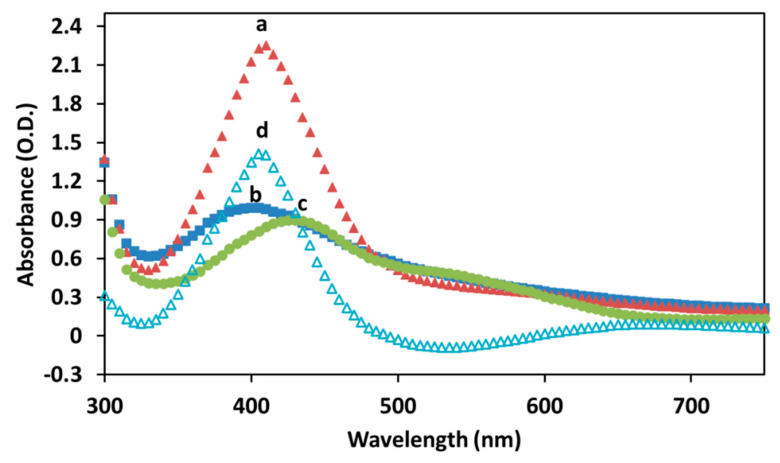
UV-visible absorption of (**a**) Cur-AgNPs before dialysis, (**b**) Cur-AgNPs after dialysis, (**c**) curcumin solution and (**d**) subtraction curve of (**a**–**c**).

Figure 6 illustrates the UV-visible spectra of the Cur-AgNPs after the synthesis (a), after the dialysis (b) and of curcumin alone (c). UV-visible spectra of AgNPs of comparable size (45 nm) reported in literature show a sharp absorption at 415 nm [28]. Cur-AgNPs instead demonstrate an absorption closer to 410 nm for the sample before the dialysis and a further blue-shift for the particles after the dialysis (400 nm). This absorption is also distinctively different when compared with that of curcumin alone (430 nm), suggesting a strong plasmonic resonance between the silver cores and the curcumin outer shell. Macroscopically, the Cur-AgNPs dispersion after the synthesis has a faint brown-yellow color, probably due to unreacted curcumin in the reaction mixture. After the dialysis the dispersion lost most of its color gaining only a light brown shade, while concentrated dispersions had a more intense brown color.

Additionally, the subtraction curve between the Cur-AgNPs dispersion after the synthesis and the spectrum of curcumin in solution is shown in Figure 6d. The resulting curve could be used to indicate recovery efficiency of NPs after dialysis (in this case 70%), assuming that the volume of the samples did not change. 

**Figure 7:**
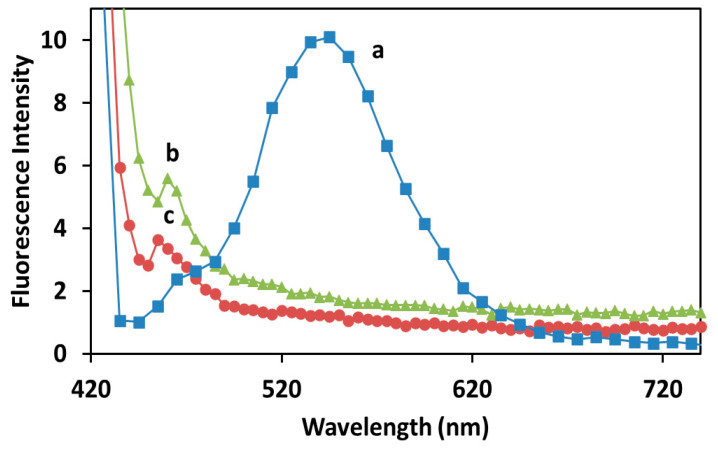
Fluorescence intensity emission of (**a**) curcumin alone, (**b**) Cur-AgNPs before dialysis and (**c**) Cur-AgNPs after dialysis. The most representative experiment among the five different batches produced with this synthesis is shown.

In addition to their UV-visible absorption, the samples were also evaluated in terms of their fluorescence spectroscopy, as shown in Figure 7.

All solutions tested exhibited an extremely weak fluorescence emission, with curcumin alone (Figure 7a) having a more intense but broad emission from 460 to 620 nm. The NPs formation resulted in the disappearance of the broader emission, keeping only a small but sharp emission at 460 nm (Figure 7b). As expected, after dialysis this peak was preserved but it was reduced to approximately the 65% of the pre-dialysis intensity (Figure 7c).

## 3. Discussion

Due to the unique anatomy and physiology of the human eye, efficient ocular drug delivery is a great challenge for researchers and pharmacologists. Although there are several noninvasive and invasive treatments, such as eye drops, injections and implants, the current treatments either suffer from low bioavailability or severe adverse ocular effects. Interestingly, the emerging nanoscience and nanotechnology are playing an important role in the development of novel therapeutic strategies for ocular diseases [21]. Here, we propose an innovative nano-formulation to solubilize and make more bioavailable a natural product derived from *Curcuma longa*, known for its in vitro apoptogenic effects on cells derived from human pterygium [5,6]. To obtain such a compound we conjugated curcumin to Ag-NPs as the nanocarrier improving curcumin bioavailability and solubility. This product was designed to respect the ocular pH and osmotic pressure and to avoid toxic effects. In fact, it has been demonstrated that curcumin has beneficial effects on several ocular diseases, such as chronic anterior uveitis, diabetic retinopathy, glaucoma, age-related macular degeneration, and dry eye syndrome [29]. On the other hand, silver nitrate has been widely used all over the world for the treatment of perinatal eye infections with no evidence of any toxicity [17]. Colloidal AgNPs have been demonstrated to be relatively safe when administered to oral mucosa, eye and skin of the animal models for short periods of time [20]. In addition, the human eye is a relatively closed organ always considered a perfect research object for drug delivery because the systemic circulation is usually omitted. Furthermore, human corneal epithelium and stroma represent a barrier that hinders passage of hydrophilic and hydrophobic molecules, respectively, and, moreover, blood-aqueous and blood-retinal barriers prevent penetration of molecules into the intraocular chamber, resulting in inefficient therapy on intraocular tissues [21]. Beside ocular barriers, clearance mechanisms of the corneal surface and other precorneal factors (eye blinking, tear film, lacrimation, etc.) reduce efficacy and bioavailability of ocular drugs. All these mechanisms guarantee the absence of toxicity of many compounds used as topic drugs for the treatment of ophthalmic diseases. Hence, the formulation will be administered as a topic remedy, for really short time intervals and at a very low dosage to avoid both ocular and systemic side effects.

In the synthesis used for this work several significant modifications of Bettini’s method have been applied [22], aiming to obtain a biocompatible and effective formulation, ready for the preparation of a prototype for human use.

To reduce the contacts between the environment of the laboratory and the reaction mixture, the synthesis procedure was carried out in a reaction flask in which the silver nitrate was injected through a septum. To further reduce the possibility of bacterial contamination entering in contact with the synthesis environment, the synthesis was performed at higher working temperature, 100 °C in reflux against 90 °C for the Bettini’s synthesis, thus reducing the time of reaction and time in which the reaction mixture was exposed to the environment. Moreover, the resulting solution was dialyzed to remove any unreacted curcumin and other residues of reaction, using a membrane with a mesh size small enough to prevent the contamination with bacteria and to prevent possible contamination with bacterial endotoxins [30]. The final advantage of this synthesis procedure was the introduction of a physiological buffer which was achieved without compromising the stability of the particle distribution, opening the way for its conversion to a formulation compatible with the administration in humans in the form of eye drops.

**Figure 8:**
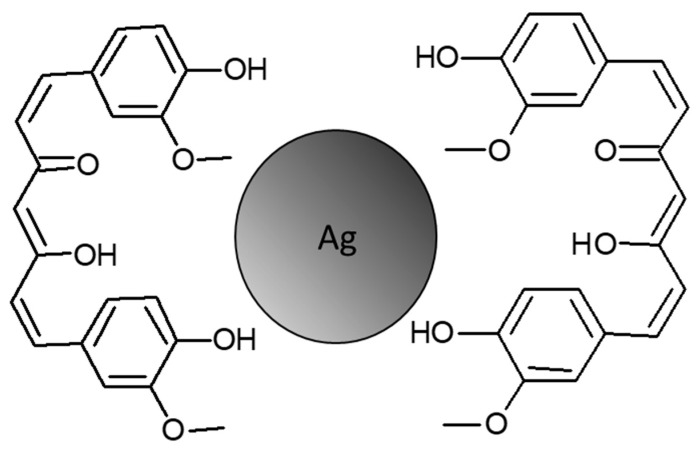
Schematic representation of the interaction between curcumin and silver NPs in Cur-AgNPs.

The quality of the synthesis in terms of reproducibility and size control was demonstrated by the analysis of the TEM pictures (Figure 1 and Figure 2) and by the study of the hydrodynamic diameters of the particles with DLS (Figure 3), while the presence of curcumin acting as a capping agent for the silver NPs (Figure 8) was demonstrated using a combination of techniques.

The first and more significant proof of the presence of curcumin on the surface of the Cur-AgNPs was the ζ-potential (Figure 4) which showed a clear negative charge on the surface of the NPs, consistent with the negative charge of carbonyl moieties at pH 7 [31].

Another clear indication for the chemical coordination of organic molecules on the surface of the NPs was obtained using FTIR (Fourier-transform infrared) spectroscopy (Figure 5): this measurement indicated that after the dialysis step, hydroxyl groups are present onto the particles surface together with C=C and C=O double bonds [32]. The appearance of a fluorescence emission peak (Figure 7) in the sample containing the particles indicates the presence of a delocalized molecule in proximity with the silver core [33]. At the same time, the quenching of the broader fluorescence emission of the phenol groups in the fluorescence spectra and of the absorption peaks in the FTIR spectrum (Figure 5b) suggests that the aromatic rings directly interact with the silver core [34].

The obtained value of the ζ-potential (Figure 4) and the sufficient interparticle distances among particles in TEM images (Figure 1) suggest that the colloidal NPs dispersions are stable. In the basic conditions of the synthesis the carbonyl and hydroxyl moieties of curcumin possessed a ζ-potential of ≈−40 mV while at pH 7 their potential was closer to −32.5 mV. However, this high value of ζ-potential still makes the Cur-AgNPs very stable [27,35].

Of course, the batches of product obtained have been tested on in vitro cultured keratinocytes derived from human pterygium explants to determine the biological efficacy of the formulation. Preliminary results showed a decreased percentage of viable cells in samples treated with Cur-AgNPs versus untreated controls (62 ± 3% vs. 100 ± 3%) (unpublished observations). Further in vitro and in vivo research in the animal model and in humans will enable us to analyze efficacy, safety, and tolerability of this innovative nano formulation.

## 4. Materials and Methods

### 4.1. Materials

Silver nitrate (≥99.0%, AgNO_3_) and sodium hydroxide (97%, NaOH) were purchased from Sigma Aldrich, Dorset, UK. Ethanol (reagent grade, EtOH) and curcumin (95%, Cur) were bought from Farmalabor, Canosa di Puglia, Italy. Dialysis tube (12 kDa MW) and borax buffer (pH 9) were obtained from Thermo Fisher, Dartford, UK.

### 4.2. Methods

#### 4.2.1. Synthesis of Cur-AgNPs

In the beginning, a small aliquot of solution of curcumin in ethanol (10.6 mM, 1 mL) was dispersed in 1 L of ultrapure water to obtain a final concentration of 10.6 μM. The pH of the solution was regulated to 8.5–9 using small aliquots of NaOH 1 M. The formation of the particles initiated when 99 mL of the curcumin solution was heated to the boiling point and a small aliquot of an AgNO_3_ (1 mM, 1 mL) solution was added under moderate stirring speed (350 rpm). The resulting solution was dialyzed against 10 mM borax buffer for 48 h changing the dialysis medium every 24 h.

#### 4.2.2. Characterization of Cur-AgNPs

The hydrodynamic diameter of the Cur-AgNPs and their ζ-potential was measured through DLS technique using a Zetasizer Nano device from Malvern Instruments, while the diameter of the silver core of the particles was measured through the statistical analysis of the TEM images acquired using a JEOL 1200 EX TEM operating with a 120 KV acceleration voltage. The presence and the composition of the curcumin shell surrounding the particles was characterized by means of UV-visible and fluorescence intensity analysis performed with SpectralMax (excitation wavelength 405 nm, attenuator 75) and confirmed with FTIR spectroscopy using a Perkin Elmer System 2000 spectrometer.

## 5. Conclusions

Nowadays, current management strategies for pterygium imply surgical excision that is a complex and invasive procedure that most often results in the recurrence of a lesion which can turn up to be more clinically aggressive than the initial one. Hence, the identification of a new potential non-invasive treatment of pterygium is of utmost importance. In this study, a simple, green, and cheap procedure has been developed to synthesize Cur-AgNPs in an exemplar formulation compatible with the development of eye drops suitable for the in vivo treatment of human pterygium. The formulation presented in this work represents an important step in the creation of an ophthalmic product able to tackle an invasive and quite severe human disease, which is now difficult to treat, let alone eliminate. Further in vitro and in vivo studies, both in animal models and in humans, will be necessary to test efficacy and tolerability of this innovative compound.

## Sample Availability

Samples of the compounds are not available from the authors.
